# Genome Mining for New Enzyme Chemistry

**DOI:** 10.1021/acscatal.3c06322

**Published:** 2024-03-12

**Authors:** Dinh T. Nguyen, Douglas A. Mitchell, Wilfred A. van der Donk

**Affiliations:** ^†^Department of Chemistry, ^‡^Carl R. Woese Institute for Genomic Biology, University of Illinois at Urbana-Champaign, Urbana, Illinois 61801, United States; § Howard Hughes Medical Institute at the University of Illinois at Urbana-Champaign, Urbana, Illinois 61801, United States

**Keywords:** genome mining, enzymes, biocatalysis, database, peptides, RiPPs, bioinformatics

## Abstract

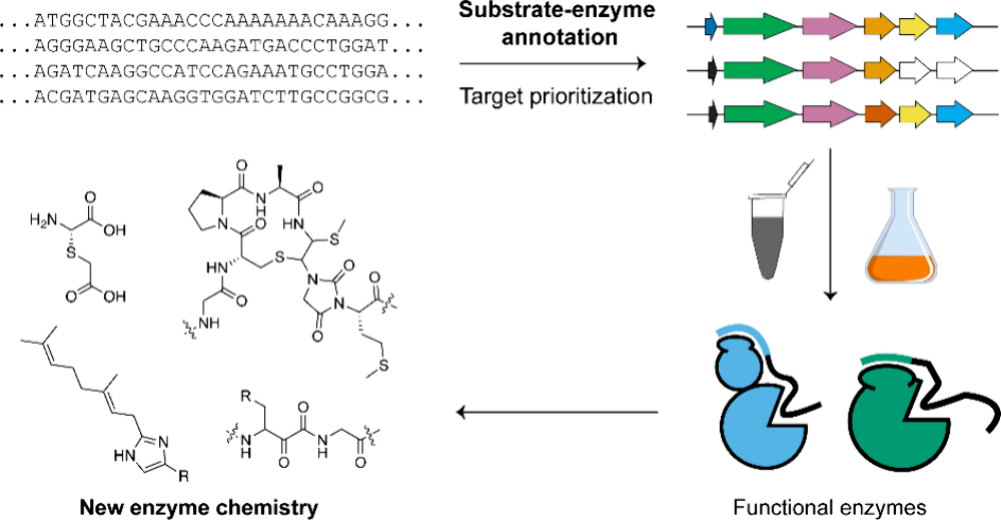

A revolution in the field of biocatalysis has enabled scalable access to compounds of high societal values using enzymes. The construction of biocatalytic routes relies on the reservoir of available enzymatic transformations. A review of uncharacterized proteins predicted from genomic sequencing projects shows that a treasure trove of enzyme chemistry awaits to be uncovered. This Review highlights enzymatic transformations discovered through various genome mining methods and showcases their potential future applications in biocatalysis.

## I Introduction

Enzymes are pivotal for biological processes and are valuable catalysts for many industrial applications.^1−3^ The ability of enzymes to catalyze complex transformations efficiently makes them invaluable for applications in agriculture, food production, consumer goods, and medicine.^1,4−6^ The use of enzymes enhances the synthesis of pharmaceuticals by improving atom economy, minimizing waste generation, and elevating the selectivity of chemical reactions.^7,8^ Additionally, enzymatic synthesis facilitates the fermentation of genetically modified organisms on the industrial scale, providing greener and more sustainable access to commodity chemicals.^9,10^

Enzymatic synthesis of a selected target typically commences with retrosynthetic analysis.^11,12^ Candidate enzymes that could facilitate the assembly of potential precursors are identified through review of scientific literature and online databases that catalog enzymatic reactions.^13−16^ Once tentative catalysts have been identified, directed evolution or other protein engineering techniques can be employed for reaction optimization.^17,18^ As has been pointed out, the scope and efficiency of biocatalytic processes would be enhanced by expanding the repertoire of available enzymatic transformations.

Exploring reactions catalyzed by uncharacterized proteins in genomic databases offers a promising path to uncover unique enzymatic activities.^19^ The rapid expansion in genome sequencing has led to the identification of a multitude of uncharacterized enzymes, either misannotated as having a function equivalent to known enzymes or annotated as hypothetical proteins or domains of unknown function (DUFs).^20^ Notably, certain enzyme superfamilies are recognized for their ability to facilitate a wide range of chemical reactions.^21−24^ Hence, elucidating new enzymatic functions from genomic data is a promising avenue for unlocking novel chemical reactions.

A notable challenge in uncovering enzyme function is identifying the appropriate substrate. One approach to predicting candidate substrates includes docking a comprehensive ligand library (composed of metabolites from biological databases) to multiple enzymes in one biosynthetic pathway.^25^ In a natural product class termed the ribosomally synthesized and post-translationally modified peptides (RiPPs),^26,27^ substrate prediction is much less computationally intensive, as these compounds are produced from a genetically encoded precursor peptide(s) that is typically encoded near other genes for the biosynthetic enzymes. Thus, any uncharacterized proteins in a biosynthetic gene cluster (BGC) will most likely act on the precursor peptide or a derivative made by the other enzyme(s) in the BGC. Heterologous expression of the gene-encoded substrate(s) with the enzymes of the BGC can therefore not only facilitate the discovery of new natural products but also assign function to uncharacterized enzymes.

**Figure 1:**
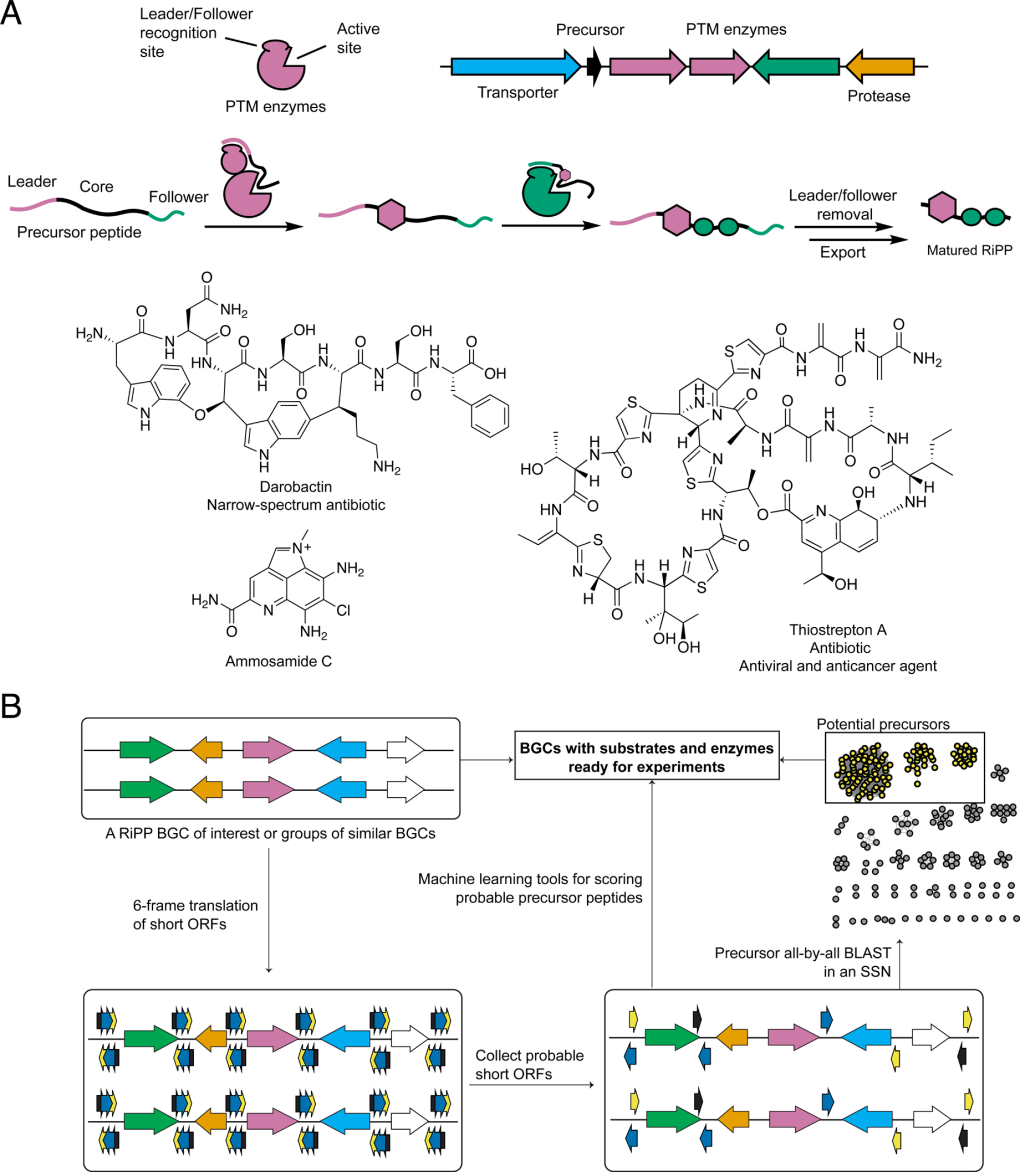
(A) General concept of RiPP biosynthesis and structures of select examples of RiPPs. (B) A general workflow describing the process of identifying putative precursor peptides in RiPP BGCs of interest.

The RiPP precursor peptide contains a core region that receives the post-translational modifications (PTMs) and an N-terminal leader region and/or a C-terminal follower region responsible for recruiting the modifying enzymes (Figure 1A).^27^ At present, more than 40 classes of RiPPs have been reported, categorized by common, class-defining PTMs. Individual members of a class may have additional, compound-specific tailoring PTMs. Examples of RiPPs include those formed by a few enzymes (e.g., darobactin, matured by a modifying enzyme and proteolysis),^28^ hypermodified peptides formed by a cascade of tailoring enzymes (e.g., thiostrepton),^29^ or even nonpeptidic small molecules (e.g., ammosamide C, Figure 1).^30^ Recent advancements in the analysis of short open-reading frames (ORFs) in prokaryotic genomes have greatly facilitated the identification of RiPP precursor peptides, both for known classes and in yet-uncharacterized families.^31−38^

A general workflow utilized in many genome mining studies to identify RiPP precursor peptides of BGCs of interest is depicted in Figure 1B. Past challenges in identifying the RiPP precursor peptide are associated with their small size, sequence diversity, and the frequent lack of predictable secondary structure. These issues are mitigated by tools like RODEO, which employs a six-frame translation of intergenic regions of a candidate BGC to identify unannotated small genes (Figure 1B).^31^ Marriage of RODEO with gene finding algorithms like Prodigal^39^ can be useful, as shown with the program RiPPER,^34^ to locate the most probable short ORFs by evaluating ribosome binding sites (RBS), start codons, and G/C content. The candidate precursor peptides are then evaluated through machine learning algorithms (such as support vector machine classification), which identify traits of precursor peptides based on characterized RiPP pathways.^31,35−37^ This task is often aided by sequence similarity networks (SSNs) and sequence alignments of the identified short ORFs, which point out the most probable precursor peptide(s) through sequence conservation.^40−44^

This Review centers on the latest advances in identifying unprecedented enzymatic functions by analyzing prokaryotic RiPP BGCs. This Review will not cover enzyme functions identified through assigning BGCs to metabolites that had been detected in extracts or metabolomic data prior to genome searches and molecules previously documented in the literature. We also will not cover enzymatic reactions that are unprecedented to RiPPs but that have been reported in other contexts or that from a chemical perspective are not surprising. Our focus is on the use of genomic analysis to characterize new enzymatic activities within the context of RiPP biosynthesis. We concentrate on two primary strategies: first, characterizing BGCs that contain homologues of known RiPP enzyme families, and second, development of bioinformatic techniques to detect new RiPP classes in an enzyme-agnostic fashion. These elements serve as markers to identify putative RiPP pathways, with a particular focus on those featuring unfamiliar enzymes and/or substrates. Notably, these methods are not necessarily mutually exclusive; orthogonal approaches can lead to the computational identification of similar BGCs.

## II Reaction Discovery via Homology to Known RiPP Enzymes

**Figure 2:**
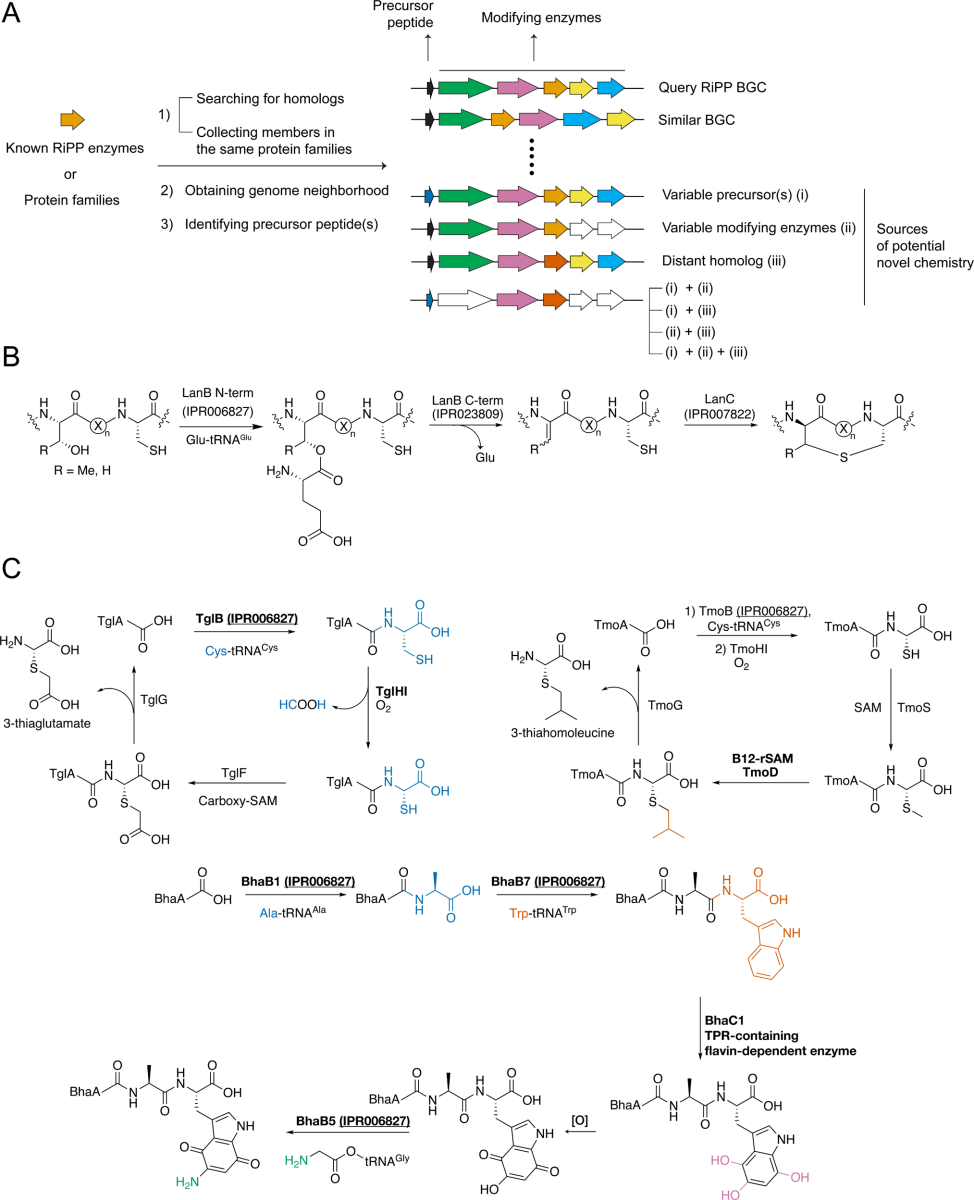
Novel RiPP enzyme chemistry resulting from homology-based genome mining. The enzymes performing novel chemistry are bolded, while the utilized bioinformatic hooks are underlined. In many cases, the IPR protein families used as the bioinformatic handle yielded novel chemistry. Colors are either used to depict bond formations or conceptually describe a BGC. (A) General concept of homology-based mining. (B) Canonical class I lanthipeptide biosynthesis. X = amino acid. (C) Pearlin biosynthetic pathways identified from mining LanB homologues. The BGCs were identified from homology searches using LanB (TglB, BhaB1, BhaB7, BhaB5), whereas the activities of TglHI-, TmoD-, and TPR-containing flavin-dependent (BhaC1) enzymes were discovered by coexpression studies taking advantage of the genetically encoded substrates and the linear biosynthetic pathways of most RiPPs.

Homology-based mining starts with retrieving homologues of known RiPP enzymes using sequence homology searches [e.g., Basic Local Alignment Search Tool (BLAST)]^45^ and/or the profile hidden Markov models (pHMMs)^46,47^ representing the RiPP enzyme families (Figure 2A). Many protein databases utilize pHMMs to catalog protein families, with InterPro (IPR)^48,49^ being the most comprehensive resource, as it is a collection of smaller pHMM libraries (e.g., Pfam^50,51^ and TIGR/NCBIFAM^51^). For consistency, this Review uses IPR identifiers to describe all discussed RiPP enzyme families. The assembled pool of putative RiPP enzymes is then subdivided into different groups based on primary structure using SSNs^41^ and/or phylogenetic trees.^52,53^ Then, the genome neighborhood of homologues of interest is collected to identify potential modifying enzymes and the precursor peptide(s). BGCs anticipated to uncover new RiPP chemistry based on uncharacterized enzymes or unusual gene combinations are usually prioritized based on several criteria (Figure 2A): (i) the presence of distant homologues of the query proteins or uncharacterized families/subfamilies within a selected superfamily; (ii) BGCs featuring substrate peptides with unusual sequences; (iii) BGCs comprising a different set of modifying enzymes compared to those containing the query proteins of known RiPP enzymes; and (iv) BGCs that exhibit more than one of these attributes.

### II.a Lanthipeptide Synthetase Homology as a Route to Discover New Enzyme Chemistry

Lanthipeptides are defined by thioether linkages resulting from a Michael-type addition of Cys residues to dehydroalanine (Dha) or dehydrobutyrine (Dhb) (Figure 2B).^54,55^ To date, researchers have identified five distinct lanthipeptide subclasses, each delineated by how the dehydroamino acids are formed and the specific features of the cyclase enzymes responsible for thioether formation. The characterization of lanthipeptide-like BGCs containing unusual features revealed new RiPP classes and enzymatic functions.

#### Pearlins

Peptide aminoacyl-transfer ribonucleic acid (tRNA) ligase-derived RiPPs (i.e., pearlins) were discovered from investigations of LanB enzymes (IPR006827). These proteins consist of an N-terminal domain that facilitates glutamyl-tRNA-dependent transesterification of the glutamyl group to the side chains of Ser or Thr residues and a C-terminal domain (IPR023809) that catalyzes an elimination reaction on these glutamylated residues to form Dha or Dhb (Figure 2B).^56^ In silico analyses identified numerous BGCs that lack a lanthionine cyclase (IPR007822) and Cys residues in the precursor peptide but that feature truncated LanB enzymes (termed short LanBs) possessing only a glutamylation-like domain.^30^

Coexpression in *Escherichia coli* of the precursor peptide and the short LanB (TglB, NCBI accession code: WP_007252217.1) of one such BGC from *Pseudomonas syringae* unveiled an unexpected new function of the LanB superfamily: ligation of an amino acid, in this case cysteine, via an amide bond to the C-terminal carboxylate of the precursor peptide (Figure 2C).^30^ Subsequent investigations on BGCs from *Bacillus halodurans* and *Streptomyces sp.* CNR-698 demonstrated the versatility of the short LanBs, with Ala and Trp also being conjugated to the C-termini of their substrate peptides.^57^ Hence, the renaming of short LanBs as peptide aminoacyl tRNA ligases (PEARLs). These discoveries introduced a biosynthetic concept distinct from other canonical natural product pathways involving the transient attachment of an amino acid to a peptide scaffold, which after extensive chemical modification, is released as a distinct small-molecule natural product—a process that contrasts with conventional RiPP PTMs resulting in modified peptides (Figure 2C).

Additional new enzymatic transformations were also uncovered in these investigations. Taking advantage of the linearity of most RiPP biosynthetic pathways, once the activity of TglB was determined, the function of the multinuclear iron-dependent oxidative enzyme (MNIO, formerly DUF692, IPR007801) TglH (WP_007252215.1) from the *P. syringae* BGC was determined by coexpression in *E. coli.* TglH together with the substrate-binding protein TglI (WP_007252216.1) catalyzes the unprecedented excision of the β-carbon of the TglB-appended Cys residue to form a C-terminal 2-amino-2-mercaptoacetate. This structure is converted by more conventional enzymatic reactions to a C-terminal 3-thiaglutamate (Figure 2C). A protease in the BGC then cleaves off this product from the scaffold peptide to allow for catalytic turnover.^30^ A related BGC (*tmo*) was found in *Tistrella mobilis* with TmoB and TmoHI catalyzing chemistry analogous to TglB/HI. The subsequent steps differ, as revealed by coexpression experiments in *E. coli.* The *tmo* BGC contains the *S*-adenosyl-l-methionine (SAM)-dependent methyltransferase (IPR029063) TmoS (WP_014744571.1) and the cobalamin- and radical *S*-adenosyl-l-methionine-dependent enzyme (B12-rSAM, IPR034466) TmoD (WP_228382265.1).^58^ TmoS is responsible for *S*-methylation of the C-terminal 2-amino-2-mercaptoacetate, while TmoD performs three successive B12-dependent *C*-methylations to form 3-thiahomoleucine (Figure 2C).

The *bha* pathway uses two PEARLs, BhaB1 (WP_161598560.1) and BhaB7 (WP_010898205.1), to attach Ala and Trp at the C-terminus of a scaffold peptide, respectively (Figure 2C). A previous protein of unknown function, BhaC1 (WP_010898191.1), was demonstrated to be a unique flavin mononucleotide-dependent enzyme with a tetratricopeptide domain (IPR011990) that introduces three hydroxyl groups to the C4, C5, and C7 positions of the ligated Trp residue.^57^ These hydroxylations set the stage for subsequent oxidation to form an *ortho*-hydroxy *para*-quinone structure. This moiety then serves as a substrate for another PEARL (BhaB5, WP_041820575.1), which attaches Gly to Trp-C5 (Figure 2C). Ultimately, the glycine adduct is transformed to an amine, revealing a heretofore unprecedented route to aromatic amines. These transformations were leveraged for the previously elusive biosynthesis of the myosin-targeting^59^ ammosamides (e.g., Figure 1A). Thus, the characterization of RiPP BGCs containing a peculiar LanB homologue revealed new biosynthetic logic and enzymatic transformations. A related observation of BGCs encoding truncated LanB enzymes but neither lanthionine cyclase(s) nor precursor peptides containing Cys residues led to the discovery of pyridine-based cyclic peptides (termed pyritides) similar to thiopeptides but without thiazol(in)es.^60,61^

#### Cyclization Reactions Forming Thioaminal, Thiomethyl, and Hydantoin Groups

**Figure 3:**
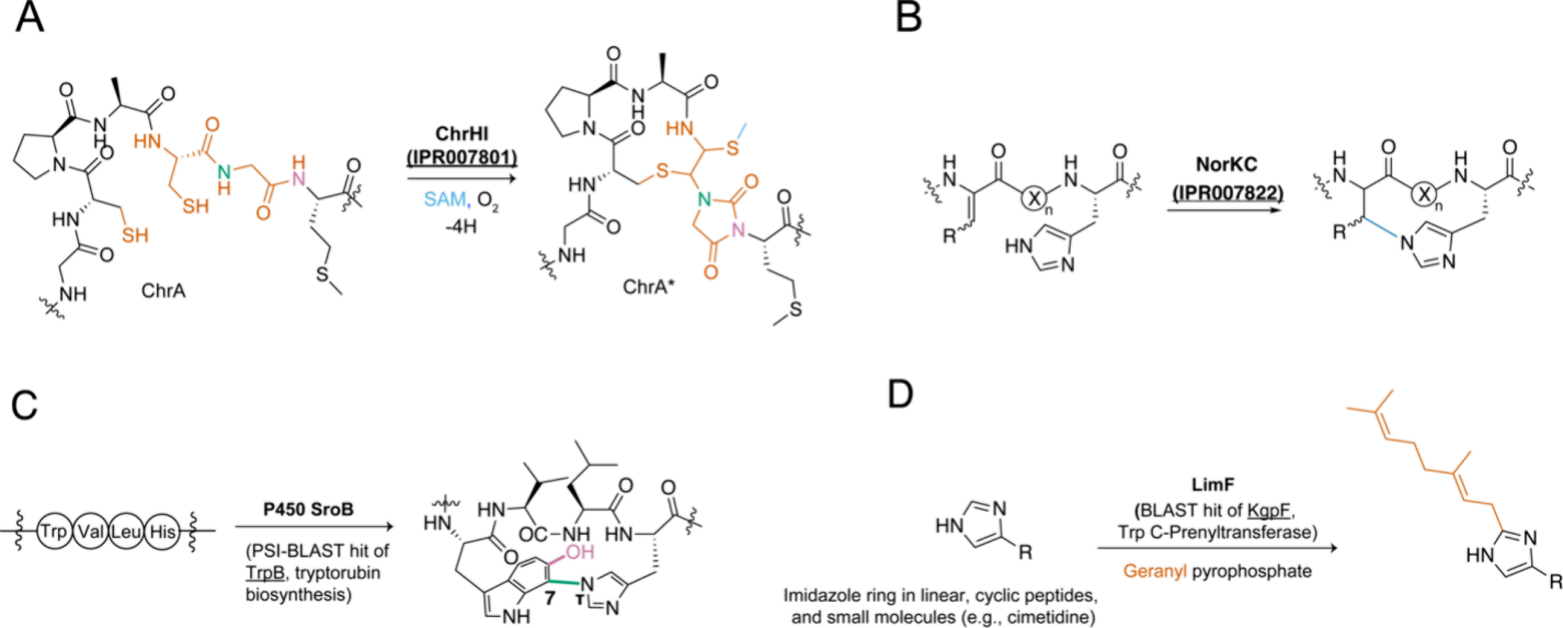
Novel RiPP enzyme chemistry resulting from homology-based genome mining. The enzymes performing novel chemistry are bolded, while the utilized bioinformatic hooks are underlined. In many cases, the IPR protein families used as the bioinformatic handle yielded novel chemistry. Colors are used to depict bond formations. (A) Enzymatic activity of the MNIO ChrH. (B) His-Dhb cross-link formation catalyzed by the class III lanthipeptide synthase NorKC. (C) C–N cross-link formation and hydroxylation catalyzed by the P450 SroB. (D) Histidine C2 geranylation catalyzed by the cyanobactin prenyltransferase LimF.

TglH was the second characterized MNIO protein after MbnB (WP_065083569.1), which acts in methanobactin biosynthesis by converting two Cys into two oxazolone/thioamide pairs in the MbnA precursor peptide.^62^ A SSN of the MNIO family (IPR007801) highlighted a subgroup predominantly from the *Chryseobacterium* genus. The corresponding BGCs encode the MNIO protein ChrH (WP_034727520.1), a putative anthrone oxygenase-like protein (IPR013901, formerly DUF1772) ChrI (WP_165571891.1), a ribulose phosphate-3-epimerase-like enzyme (IPR000056) ChrE (WP_034727525.1), and an M42 peptidase (IPR008007) ChrP (WP_034727527.1).^63^ The substrate peptide (ChrA) contains a conserved CPACGMG sequence at the C-terminus. Coexpression of ChrA with ChrHI in *E. coli* resulted in a macrocycle with two thioaminals and a thiomethyl group, and a five-membered imidazolidinedione (hydantoin, Figure 3A). The overall process was without precedent, with the only similarity to reactions catalyzed by other MNIOs being the net four-electron oxidation and chemistry involving Cys. ChrHI uses SAM for methylation of the thiol of a thiohemiaminal (Figure 3A). The predicted structures of ChrH and ChrI (AlphaFold)^64^ do not exhibit the typical Rossmann fold for SAM binding, suggesting a new set of residues comprise the ligand binding pocket.^63^

All characterized MNIOs (MbnB, TglH, ChrH) are iron-dependent. The crystal structures of MbnBC and TglHI revealed two to three irons in the active sites of MbnB and TglH.^65−67^ The crystal structure of MbnBC bound to MbnA,^66^ the AlphaFold model of TglHI with the precursor TglA, and biochemical assays demonstrated that MbnC (WP_003614727.1) and TglI engage their cognate precursor peptides.^67,68^ In the MbnABC structure, one Cys residue of MbnA is ligated to an iron. Comprehensive activity assays of MbnBC under varying redox conditions, complemented by electron paramagnetic resonance (EPR) and Mössbauer spectroscopy, suggested MbnB’s reliance on a mixed iron oxidation state [Fe(II) and Fe(III)] for functionality.^65^ The unique constellation of irons in these enzymes, combined with the distinct activities of ChrHI, MbnBC, and TglHI, prompted the renaming of DUF692 to the more descriptive MNIO.^63^

#### Histidine-Dehydrobutyrine Cross-Link

A RiPP displaying an unusual His-Dhb (Hbt) cross-link was discovered during genome mining efforts for new class III lanthipeptides using the class-defining lanthipeptide synthetase LanKC (IPR000719 and IPR007822). A putative class III lanthipeptide BGC (*nor*) was identified in *Streptomyces noursei* ATCC 11455, with the precursor peptide NorA containing three Ser/Thr and only one Cys.^69^ Heterologous expression in *Streptomyces lividans* and *in vitro* reconstitution demonstrated that the lanthipeptide synthetase NorKC (ANZ21440.1) catalyzes the dehydration of all three Ser/Thr to the corresponding Dha/Dhb as well as the formation of a canonical labionin between the Cys and Dha residues. Unexpectedly, NorKC also formed an unprecedented cross-link between the N_τ_ of His and C_β_ of an aminobutyric acid residue via a Michael addition of His to dehydrobutyrine (Figure 3B). Notably, the resulting lanthipeptide, termed noursin, possessed selective copper-binding activity that is dependent on the Hbt cross-link.

### II.b Cytochrome P450 Homology as a Route to Discover New Enzyme Chemistry

Cytochromes P450 (IPR001128) comprise a superfamily of heme-dependent enzymes catalyzing diverse and remarkable transformations.^24^ Besides their natural catalytic activity, directed evolution techniques have been extensively employed to engineer P450 enzymes to catalyze unnatural transformations.^17^ In RiPP biosynthesis, P450-catalyzed reactions are frequently observed, with most identified retrospectively after traditional natural product isolation efforts.^70,71^ We focus here on noncanonical cytochrome P450-catalyzed transformations. Examples include macrocyclization during the biosynthesis of tryptorubin,^72,73^ cittilins,^74^ biarylitides,^75^ and the recently discovered cihunamides,^76^ which display a C–N bond between C7 in Trp (residue i) and the N1 of another Trp (i+3). To gather additional P450s associated with RiPP biosynthesis, the P450 TrpB^72^ (WP_093582223.1) responsible for tryptorubin biosynthesis was recently utilized as a bioinformatic hook.^77^ Examination of P450-containing BGCs containing specific patterns of aromatic residues in the precursor peptides led to the discovery of SroB (WP_157848581.1) that catalyzes the formation of a C–N linkage between C7 in Trp (residue i) and N_τ_ in His (i+3) and hydroxylation of C6 in Trp (Figure 3C). The authors also identified a C–C linkage between C7 of Trp and C3 of Tyr(i+3). While a similar linkage is known from TMC-95A,^78^ the difference is that TMC-95A possesses a smaller ring size, and the C–C bond is formed in reverse residue order (Tyr_i_-Trp_i+3_). Another P450 identified in the same study catalyzed C–N bond formation between C6 of Tyr (residue i) and N1 of Trp (i+2). This specific linkage type is also observed in tryptorubin and nocapeptin A^79^ but again in a reversed residue order and smaller ring size (Trp_i_-Tyr_i+1_). Other new P450-catalyzed transformations, discovered using a substrate-centric approach, are described later in this Review (section III.c).

### II.c Prenyltransferase Homology as a Route to Discover New Enzyme Chemistry

Cyanobactins are RiPPs produced in diverse cyanobacterial taxa.^80^ Initially, all known cyanobactins were head-to-tail-cyclized; however, several linear versions have been described. Cyanobactins also undergo other modifications, such as YcaO-catalyzed azol(in)e formation, methylation, and prenylation.^80^ The cyanobactin prenyltransferases (IPR031037) perform diverse prenylation reactions with a wide variety of donor nucleophiles and prenyl acceptors.^81^ Prenylation is beneficial from the viewpoint of peptide engineering, as it can enhance the interaction of peptides with membranes resulting in increased membrane permeability and bioavailability.^82,83^

New geranylation chemistry involving C2 of His was discovered through genome mining for uncharacterized homologues of cyanobactin prenyltransferases. An SSN constructed from homologues of the Trp *C*-prenyltransferase KgpF (WP_061432709.1)^84^ retrieved by BLAST identified a unique putative prenyltransferase (LimF, RFP52075.1) from *Limnothrix* sp. CACIAM 69d, which did not group with known prenyltransferases.^85^ Furthermore, when compared phylogenetically with characterized prenyltransferases, LimF constituted a separate branch between the Tyr-O-prenyltransferase and Trp-C/N prenyltransferase groups. *In vitro* reconstitution of LimF with the predicted head-to-tail-cyclized cyanobactin substrate unveiled a novel enzymatic activity: geranylation of C2 of the His imidazole ring (Figure 3D). LimF is a bifunctional prenyltransferase, as it can also geranylate the phenolic oxygen of Tyr. Notably, LimF possesses broad substrate scope, accepting macrocyclic peptides with diverse sequences, ring sizes, and small molecules.

### II.d Radical SAM Homology as a Route to Discover New Enzyme Chemistry

The rSAM superfamily (IPR007197), currently comprised of over >800 000 members, is recognized for catalyzing a spectrum of extraordinary transformations across various metabolite classes.^23^ Despite their functional diversity, canonical rSAM enzymes generate a 5′-deoxyadenosyl radical via the reductive cleavage of SAM. Scission of the key C–S bond is afforded by electron donation into its σ* orbital from a ubiquitous [4Fe-4S]^+^ center. The 5′-deoxyadenosyl radical then typically abstracts a hydrogen atom from the substrate, yielding 5′-deoxyadenosine as a byproduct and a substrate-based radical that can undergo a range of transformations.^86^ Numerous studies have identified rSAMs with a wide spectrum of functions in RiPP pathways,^87^ which have been used for homology searching and to construct pHMMs to retrieve additional rSAM enzymes involved in peptide and protein modification.

Many RiPP-associated rSAM enzymes contain a second protein domain identified by IPR023885, which contains two additional [Fe–S] centers. Such domains are referred to as SPASM, given they are involved in the biosynthesis of Subtilosin A, Pyrroloquinoline quinone, Anaerobic Sulfatase, and Mycofactocin.^88^ Various new enzyme functions were discovered by characterizing BGCs containing orphan rSAMs with the SPASM/twitch domain (with twitch being a truncated SPASM domain that binds to one auxiliary [Fe–S] center)^89,90^ and/or homologues of known rSAMs.

#### Spliceotides

**Figure 4:**
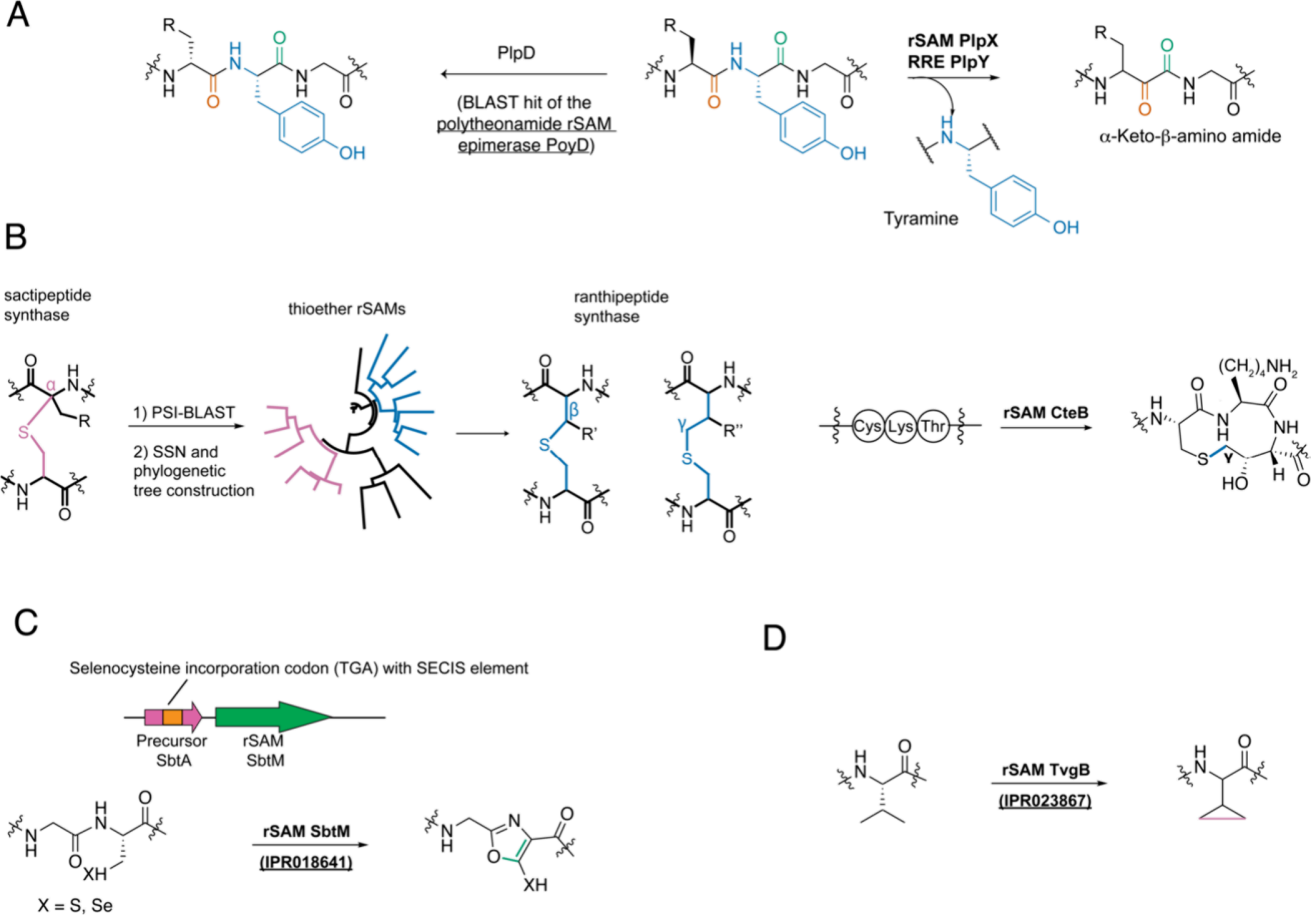
Novel enzymatic chemistry resulting from homology-based genome mining using rSAMs. (A) Tyramine splicing catalyzed by the rSAM PlpX and the RRE protein PlpY. The bioinformatic hook was another rSAM in the BGC, PlpD (homologue of polytheonamide epimerase). (B) Enzymatic activity of the ranthipeptide synthase CteB, and how ranthipeptide synthases were discovered from searching for homologues of sactipeptide synthases. (C) Seleno(thio)oxazole formation by the rSAM SbtM. (D) Cyclopropyl formation catalyzed by the rSAM TvgB.

The discovery of spliceotides resulted from exploring a BGC in *Pleurocapsa* sp. PCC 7319 (*plp*) encoding a member (PlpX) of an uncharacterized subclass of SPASM-containing rSAM enzymes (IPR026482) found alongside known rSAM epimerases. Such BGCs were first identified by BLAST searches using the rSAM epimerase PoyD (AFS60640.1) from polytheonamide biosynthesis as query. The homologue PlpD was confirmed to convert L-amino acids into D-amino acids in its peptide substrate (Figure 4A).^91^ The BGC accommodating PlpD (WP_019503883.1) and PlpX also encodes a truncated version of a RiPP precursor recognition element (RRE)^92^ and signature precursor peptides containing leader regions with similarity to either nitrile hydratase or Nif11 proteins.^93^ The function of the uncharacterized rSAM PlpX (WP_019503880.1) was elucidated by coexpression with the precursor peptides and the RRE PlpY (WP_019503879.1). PlpX was found to catalyze the unprecedented excision of tyramine from a Xaa–Tyr–Gly motif and reconnection of the peptide with an α-keto amide in the backbone, giving rise to the term spliceotide (Figure 4A).^94^ This post-translational modification is widespread in nature and has been leveraged as a bioorthogonal labeling method and moiety for protease inhibition.^95,96^

#### Ranthipeptides

Numerous RiPPs contain thioether-based macrocycles as the class-defining modification, including lanthipeptides, sactipeptides, and the more recently bioinformatically discovered ranthipeptides. While lanthipeptides are biosynthesized using Michael-like addition chemistry, sactipeptides and ranthipeptides are formed by rSAM enzymes. Sactipeptides contain a sactionine (sulfur-to-α-carbon) cross-link between a donor Cys and an acceptor amino acid, often forming a hairpin-like structure.^97^ Ranthipeptides instead contain ranthionine (radical non-α) cross-links, which are more stable relative to the isomeric sactionine.

Prior to the discovery of the first ranthipeptide, RODEO was trained to detect sactipeptides. Several rounds of BLAST and RODEO analysis of rSAMs from known (AlbA, WP_003242599.1; SkfB, NP_388073.2; ThnB, ACY56718.1; and TrnC, AED99784.1) and assumed sactipeptide BGCs (CteB, WP_003517268.1 and Tte1186, AAM24417.1)^98^ retrieved nearly 5000 proteins encoded next to high-scoring, Cys-rich precursor peptides. Sequence analysis of the rSAMs revealed a large separation between the known and assumed sactionine synthases with the latter being much more similar to QhpD (SDJ52620.1). This rSAM is responsible for the Cys–Asp (S–C_β_) and Cys-Glu (S–C_γ_) cross-links found in quinohemoprotein amine dehydrogenase.^99^ Hence, rSAMs that grouped with QhpD might not be forming sactionine linkages. This reasoning led to the discovery of freyrasin, which contains six thioether macrocycles formed by S–C_β_ linkages between Cys and Asp residues. The freyrasin synthase PapB (WP_019688962.1) possesses broad substrate scope, generating unnatural ranthipeptides with diverse ring sizes and forming S–Cγ linkages between Cys and Glu and cross-links between nonproteogenic thiols and non-natural amino acids.^100−103^ The RiPP thermocellin, previously thought to be a sactipeptide, contains a novel S–C_γ_ linkage between Cys and Thr residues formed by the rSAM CteB. These insights led to a new class of RiPPs termed radical-non-α-carbon thioether-containing peptides (ranthipeptides) (Figure 4B).^98^ Another separate ranthipeptide with the S–C_β_ linkage between Cys and Asn residues from *Streptococcus orisratti* and *Streptococcus porci* was independently discovered through genome mining for rSAMs in the neighborhood of a quorum-sensing (QS) system, which is described later in this review.^104^

#### Thio(seleno)oxazole-Containing RiPPs

A separate group of rSAM-SPASM proteins are defined by IPR018641, which contain the selenocysteine (SeCys) insertion element in their BGCs.^88^ This element is encoded immediately downstream of the TGA codon in the predicted precursor peptide, suggesting the presence of selenocysteine. The rSAM SbtM (WP_083768621) was used for *in vitro* assays with substrates containing Cys or SeCys.^105^ High-resolution mass spectral analyses demonstrated that Cys- and SeCys-containing substrate peptides lost four hydrogens after rSAM treatment. NMR analyses on the product of the Cys-substrate revealed a thiooxazole moiety (Figure 4C).

#### Polycyclopropylglycine-Containing Peptides

The discovery of a polycyclopropylglycine-containing peptide was enabled by the analysis of the SPASM domain containing anaerobic sulfatase-maturase rSAM subfamily (IPR023867), which includes characterized and predicted peptide-modifying enzymes.^88^ A BGC from *Halomonas anticariensis* DSM 16096 encodes a precursor peptide with Thr–Val–Gly–Gly repeats (TvgA), an rSAM (TvgB, WP_016417470.1), an iron- and α-keto-glutarate-dependent enzyme (IPR018724) (TvgC, WP_081645852.1), and two membrane-associated transporters (TvgD/E, WP_016417468.1 and WP_016417467.1). TvgB catalyzed the formation of multiple cyclopropyl groups by cross-linking the two methyl groups of the Val side chain in the repeat sequence (Figure 4D).^106^

#### Triceptides

**Figure 5:**
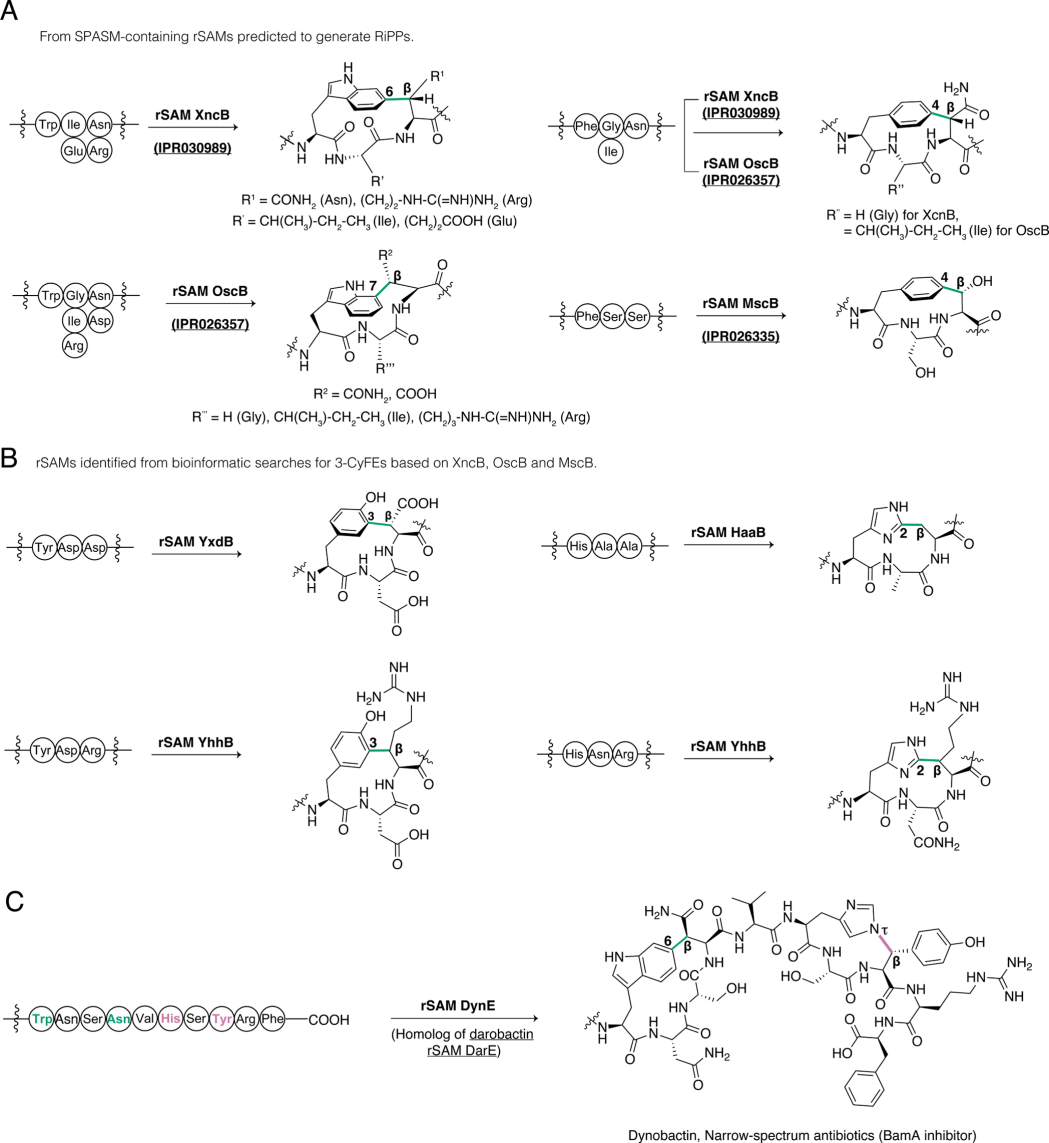
Unique enzymatic chemistry forming cyclophanes discovered by homology-based genome mining using rSAMs. (A) Novel C–C bond formation catalyzed by the 3-CyFE rSAMs XncB, OscB, and MscB. (B) Bioinformatic mapping based BLAST hits of XncB, OscB, and MscB and SPASM/Twitch-containing rSAMs from radicalSAM.org lead to novel C–C bond formation catalyzed by the 3-CyFE rSAMs YxdB, HaaB, and YhhB. (C) Discovery of a novel narrow-spectrum antibiotic with a C–C cross-link and a C–N cross-link formed by the rSAM DynE, which was identified through BLAST searches with the darobactin rSAM DarE.

Triceptides were discovered by analyzing a subset of BGCs housing the uncharacterized SPASM-containing rSAMs XncB (IPR030989, WP_010848442.1), OscB (IPR026357, OIP69445.1), and MscB (IPR026335, WP_013477425.1) and unique precursor peptides featuring conserved motifs not previously observed in reported RiPPs matured by rSAMs.^88^ These enzymes were coexpressed with the corresponding predicted precursor peptides in *E. coli*, and 2D-NMR analysis demonstrated C–C cross-links that result in three-residue cyclophanes (Figure 5A).^107^ These cross-links are located between residues i and (i+2). The salient features of these cyclophanes include: (i) XncB-catalyzed linkages between C6 (Trp) and C_β_ (Arg or Asn), (ii) OscB-catalyzed cross-links between C7 (Trp) and C_β_ (Asn or Asp), (iii) XncB- or OscB-catalyzed linkage between C4 (Phe) and C_β_ (Asn), and (iv) MscB-catalyzed bond formation between C4 (Phe) and C_β_ (Ser). This class of natural product was termed the triceptides, and the rSAMs are termed three-residue cyclophane forming enzymes (3-CyFEs).^108^

Combination of BLAST results of characterized 3-CyFEs with putative 3-CyFEs sequences identified from RadicalSAM.org, a web resource consisting of SSNs and annotation of all predicted rSAMs from UniProt and European Nucleotide Archive databases,^86^ was utilized to discover novel enzyme functions. Heterologous expression of candidate BGCs in *E. coli* followed by NMR characterization identified triceptides with a variety of rSAM-catalyzed C–C cross-links, including the formation of a C3(Tyr)–C_β_(Asp) linkage by YxdB (WP_226048651.1), generation of a C2(His)–C_β_(Ala) bond by HaaB (WP_133931326.1), and cross-linking of C3(Tyr) to C_β_(Arg) and C2(His) to C_β_(Arg) by YhhB (WP_013448981.1) (Figure 5B).^108^

#### Dynobactin

Dynobactin was discovered from a BLAST search on the rSAM DarE (WP_152962146.1), which installs C–O and C–C cross-links to form the fused bicyclic structure of darobactin (Figure 1A).^28^ Darobactin binds to the outer membrane insertase BamA and inhibits the action of the β-barrel assembly machinery (BAM) complex. RiPPER (a combination of RODEO with Prodigal)^34^ and SSN analysis were used to obtain the precursor peptides of the rSAMs retrieved from the search. A phylogenetic tree was constructed, and outgroups containing DarE-like enzymes but associated with substrates distinct from the darobactin precursor peptide were prioritized. A total of 14 strains containing the bioinformatically prioritized rSAMs were then cultivated, and the extracts were screened for growth-suppressive activity toward Proteobacteria. Bioactivity-guided isolation identified dynobactin from *Photorhabdus australis*, which contained a C–C bond between C6 of Trp_i_ and C_β_ of Asn_i+3_ and a C–N cross-link between C_β_ of Tyr_i_ and N_τ_ of His_i+2_ formed by the rSAM DynE (WP_152962146.1) (Figure 5C).^109^ In contrast to the fused bicyclic structure of darobactin (Figure 1A), dynobactin possesses two discrete macrocycles. Dynobactin also inhibits BamA with a binding mode that largely overlaps with that of darobactin.

## III Using Prevalent Elements in RiPP Biosynthesis as Bioinformatic Handles

### III.a RRE Domains as a Route to Discover New Enzyme Chemistry

The RiPP precursor recognition element (RRE) is a protein domain found in approximately half of the known prokaryotic RiPP classes.^92^ Canonical RREs are comprised of three alpha helices and three beta strands. Despite the high level of sequence diversity associated with RRE domains, the remote homology detection tool HHpred^110,111^ is capable of their retrieval. Functionally, the leader region of RiPP precursor peptides engages the RRE as a fourth beta strand, thus “completing” the tertiary fold of the RRE domain. The often nanomolar interaction between RREs and their cognate precursor peptides is critical for RiPP biosynthesis.^27^ It should be noted, however, that other putative peptide-binding domains (e.g., MbnC) do not have detectable homology with RRE domains, as defined above.

**Figure 6:**
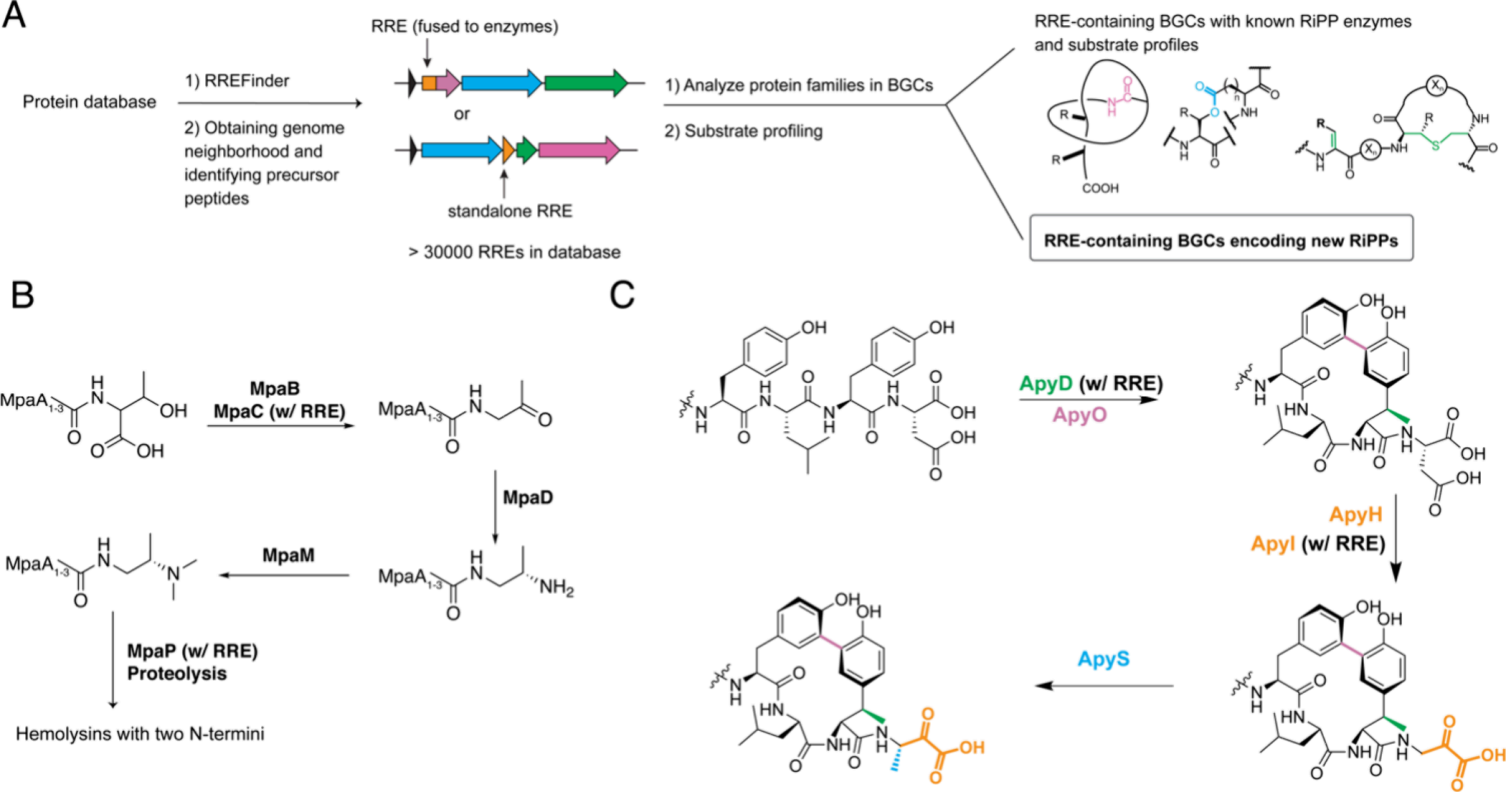
Unique enzyme chemistry discovered from mining RiPP pathways based on the RiPP precursor recognition element (RRE). (A) A general workflow discovering novel RiPP BGCs using the RRE as the bioinformatic hook. (B) The biosynthesis of daptides, a novel RiPP class identified by mining for uncharacterized RRE-containing RiPP BGCs. (C) The biosynthesis of aminopyruvatides, a new RiPP class biosynthesized by a B12-rSAM (ApyD), cytochrome P450 (ApyO), MNIO (ApyHI), and methyltransferase (ApyS) identified by mining for uncharacterized RRE-containing RiPP BGCs.

RREs are found in various RiPP classes, each having a distinct set of modifying enzymes, precursor peptide sequences, and biological functions.^92^ Consequently, RREs serve as valuable bioinformatic markers for identifying potential RiPP BGCs that may exhibit novel enzymatic activities. However, *in silico* identification of RREs presents challenges due to their considerable sequence variability and relatively small size (80–90 residues). As alluded to above, HMM-based retrieval is preferrable over a contiguous homology-based tool like BLAST for detecting remote homology. However, tools like HHpred are relatively slow and not well suited for processing many thousands of protein sequences that would be needed for even a minimal genome mining effort. To solve these issues, RRE-Finder employs a panel of custom pHMMs and secondary structure prediction using a truncated HHpred workflow. This tool identified over 30 000 RREs from UniProt.^112^ Analysis of RRE-containing BGCs revealed both BGCs encoding known and yet-uncharacterized RiPP classes (Figure 6A). Focus on BGCs containing unique substrate profiles and unprecedented combinations of modifying enzymes, followed by experimental characterization, led to the first-in-class discoveries of the daptides^38^ and aminopyruvatides.^113^

#### Daptides

The identification of daptides commenced with a comprehensive analysis of putative RRE domains identified by RRE-Finder. An SSN-guided approach classified the results into distinct RRE families. For each putative RRE, short ORFs associated with the corresponding putative RiPP BGC were identified using RODEO and Prodigal. These ORFs were then subjected to an all-by-all BLASTP within a similar SSN framework. A custom script iteratively adjusted the fractionation threshold in each SSN, which addressed a common problem in genomic enzymology where a single similarity threshold is insufficient for identifying isofunctional families. This script aimed to align the number of proteins in each RRE cluster (m) with the size of the largest SSN group from its corresponding precursor peptide SSN (n) until m ≈ n or m < n. The goal was to assign each member of a given RRE group at least one precursor peptide. After the suitable fractionation threshold for each SSN was identified, sequence alignments for each group were generated, which identified conserved motifs.^38^

This method identified a unique set of genes co-occurring with precursor peptides with unusual amino acid compositions. The examined BGC from *Microbacterium paraoxydans* DSM 15019 encompassed three precursor peptides MpaA1-A3, the RRE-peptidase (IPR008915) fusion MpaP (WP_060922558.1), the aminotransferase (IPR005814) MpaD (WP_060922561.1), the methyltransferase (IPR029063) MpaM (WP_060922559.1), the iron-dependent alcohol dehydrogenase (IPR001670) MpaC (WP_060922560.1), and the DUF-RRE fusion MpaB (WP_060922562.1). An invariant C-terminal Thr residue in the precursor peptides was determined to be the exclusive site for enzymatic modification (Figure 6B).^38^ The process involved MpaB and MpaC performing oxidative decarboxylation of the C-terminal Thr to a methylketone. Subsequently, MpaD converted the ketone into an amine, which was dimethylated by MpaM. MpaP removed the leader peptide, resulting in the mature product that exhibited helical structures and hemolytic activity. Given the conversion of the C-terminus to (*S*)-*N*_2_,*N*_2_-dimethyl-1,2-propanediamine, the new RiPP class, termed daptides, are ribosomal peptides with two N-termini.^38^

#### Aminopyruvatides

RRE-Finder identified a set of BGCs featuring several metalloenzyme superfamilies recognized for catalyzing a wide range of complex chemical transformations. These BGCs encoded a precursor peptide, cytochrome P450, MNIO, MNIO partner protein, methyltransferase, and a B12-rSAM.^113^ RRE domains were fused to the MNIO partner protein and the B12-rSAM. Sequence alignment of the predicted precursor peptides highlighted distinct profiles, including a conserved C-terminal Asp and two aromatic residues (either Trp or Tyr) in the second and fourth residues from the C-terminus that were only present in BGCs encoding cytochrome P450 proteins.

Functional expression of the *Burkholderia thailandensis* biosynthetic pathway in *Burkholderia* sp. FERM BP-3421^114,115^ unveiled that the MNIO enzyme ApyH (ABC35712.1) and its partner protein ApyI (ABC34269.1) catalyzed the unprecedented conversion of a C-terminal Asp residue into a C-terminal aminopyruvate (Figure 6C). Hence, ApyHI represents the first instance of an MNIO modifying a non-Cys residue.^113^ The B12-rSAM enzyme ApyD (ABC34219.1) methylated the β-carbon of Tyr while the cytochrome P450 ApyO (ABC35200.1) formed a biaryl C–C cross-link between the *ortho* positions of the Tyr residues. While similar P450 reactions have been observed in non-RiPP pathways such as arylomycin^116^ and mycocyclosin,^117^ ApyO introduced an *R* axial chirality in the biaryl rings, a feature distinct from the stereochemistry observed in arylomycin and mycocyclosin.^118,119^ Given the conservation of the MNIO enzyme and the Asp-containing precursor peptide, the aminopyruvate moiety emerged as the defining motif of this RiPP class, which was termed the aminopyruvatides (Apy).^113^ Another MNIO that does not modify a Cys was recently reported from a BGC that encodes two such enzymes. One MNIO performs the same chemistry on Cys as found in methanobactins (i.e., as MbnBC), but the second MNIO catalyzes cleavage of the N–Cα bond of an Asn residue to generate a C-terminal amide.^120^

### III.b Quorum-Sensing Factor and Transporters to Identify New Enzyme Chemistry

A surge of new biochemical reactions catalyzed by rSAM enzymes have been recently reported for RiPP and non-RiPP pathways.^89^ Consequently, additional bioinformatic handles have been leveraged to discover new enzymatic reactions involving these versatile biocatalysts.

**Figure 7:**
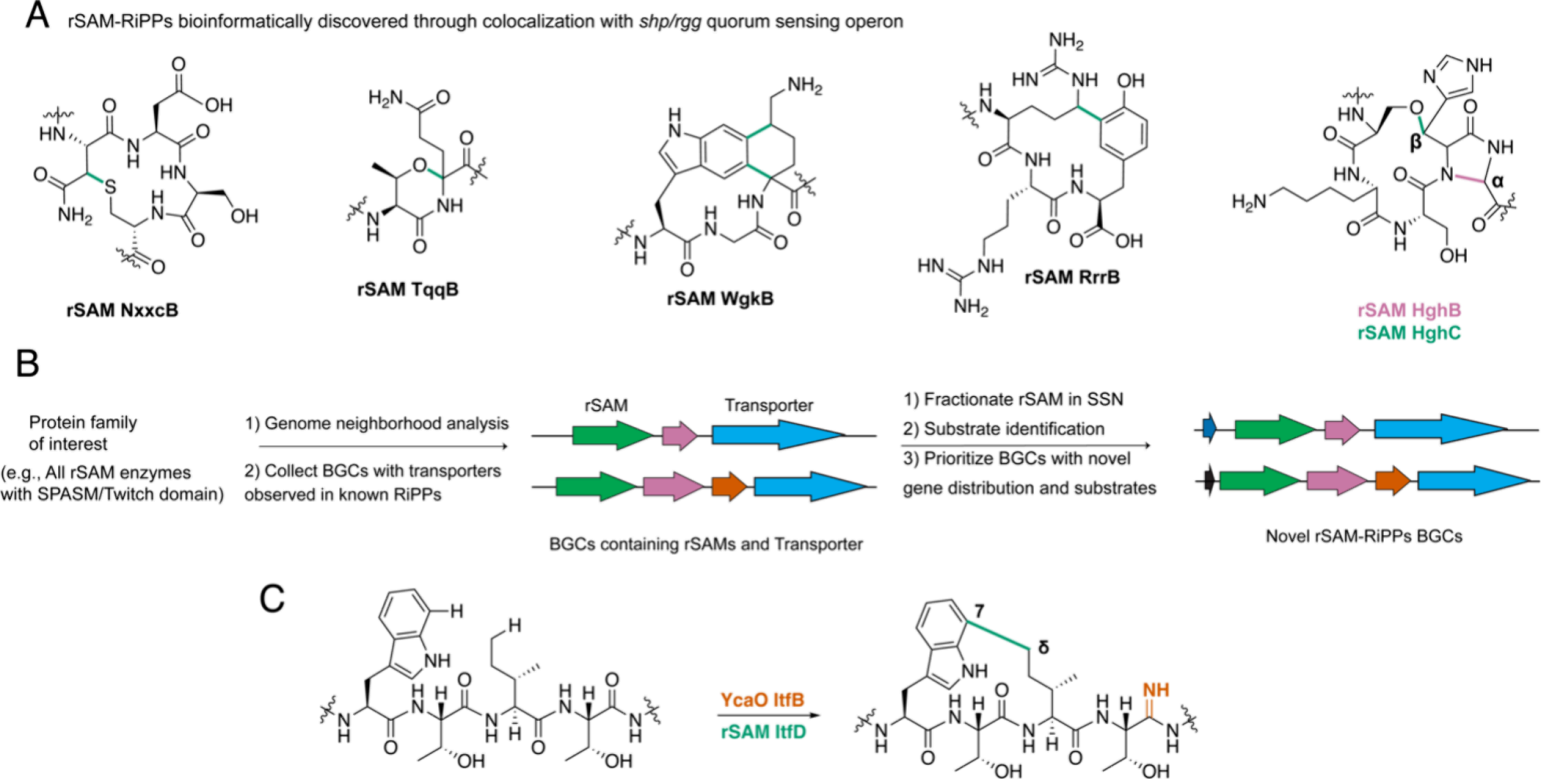
Enzyme chemistry discovered by mining quorum-sensing controlled RiPP pathways. (A) rSAM-catalyzed transformations discovered through RiPP pathways controlled by the *shp/rgg* quorum-sensing operon. (B) A general description of identifying novel RiPP BGCs utilizing transporter protein families observed in known RiPP BGCs as the bioinformatic handle. (C) Unique rSAM- and YcaO-catalyzed activity discovered in an RiPP pathway identified through the strategy depicted in (B).

The use of quorum-sensing (QS) factors as a biomarker for RiPP rSAM mining was inspired by the investigation of streptide biosynthesis in *Streptococcus*.^121^ The QS system for streptide production is an operon containing the short hydrophobic peptide (SHP) and a transcriptional regulator (RGG) located upstream of the streptide BGC. SHP, a pheromone, is exported by a subset of cells and later imported once an extracellular threshold concentration has been produced.^122^ Intracellular SHP then binds to its cognate RGG, which activates streptide production. Streptide possesses a C–C cross-link between C_β_ of Lys (residue 2) and C7 of Trp (residue 6), which is formed by the rSAM StrB (ABJ66529.1).^121^ As SHP/RGG has been shown to regulate other processes in *Streptococcus*, including other streptide-like pathways,^123^ the identification of distinct rSAM and peptide substrates downstream of the *shp/rgg* operon was anticipated to potentially lead to novel structures of rSAM-modified RiPPs. Approximately 600 rSAMs in *Streptococcus* were found to be encoded near substrate peptides and controlled by *shp/rgg*.^104^ Various new rSAM enzymatic activities (Figure 7A)^104,124−126^ were discovered with this method and were recently reviewed.^127^ The most recent pathway discovered was that of bicyclostreptin.^128^ Specifically, a BGC containing two rSAM enzymes HghB (WP_283265752.1) and HghC (WP_017649765.1), the RRE protein HghD (WP_017649764.1), and a precursor peptide (HghA) with a conserved C-terminal His–Gly–His motif was characterized. First, the rSAM HghC catalyzed formation of an O–C cross-link between the hydroxy group of Ser_i_ and C_β_ of His_i+3_ (Figure 7A). Then, HghB, which required HghC-modified HghA, catalyzed a N–C cross-link between the N–H amide of His_i_ and C_α_ of Gly_i+1_ to form a five-membered cyclic moiety.

Prevalent genes found in RiPP BGCs encode for ABC transporters, which export the RiPP upon maturation.^27^ As the QS SHP/RGG operons are only conserved in *Streptococcus*, the same research group expanded their rSAM mining by querying for SPASM/twitch-containing rSAM that co-occur with transporter-encoding genes (Figure 7B).^129^ Short ORFs in the genome neighborhood were then identified, and precursor peptide SSNs were constructed. This procedure identified a BGC from *Bacteroides thetaiotaomicron* containing the rSAM ItfD (WP_004319392.1), the ATP-dependent backbone-activating YcaO-like protein (IPR003776) ItfB (WP_004319383.1) fused to an [Fe–S]-recognition domain, the ABC transporter/C39 peptidase ItfE (WP_044917720.1), the TonB-dependent transporter ItfF (WP_004319394.1), and the precursor peptide ItfA with a conserved C-terminal Ile–Thr–Phe motif. Structural analyses after heterologous expression in *E. coli* revealed that ItfB installed a backbone amidine using NH_4_Cl as the nitrogen source, and ItfD catalyzed an unprecedented C–C cross-link between C7 of Trp_i_ and the unactivated primary carbon C_δ_ of Ile_i+2_ (Figure 7C).

### III.c Precursor Peptide(s) as the Route for Identifying New Enzyme Chemistry

**Figure 8:**
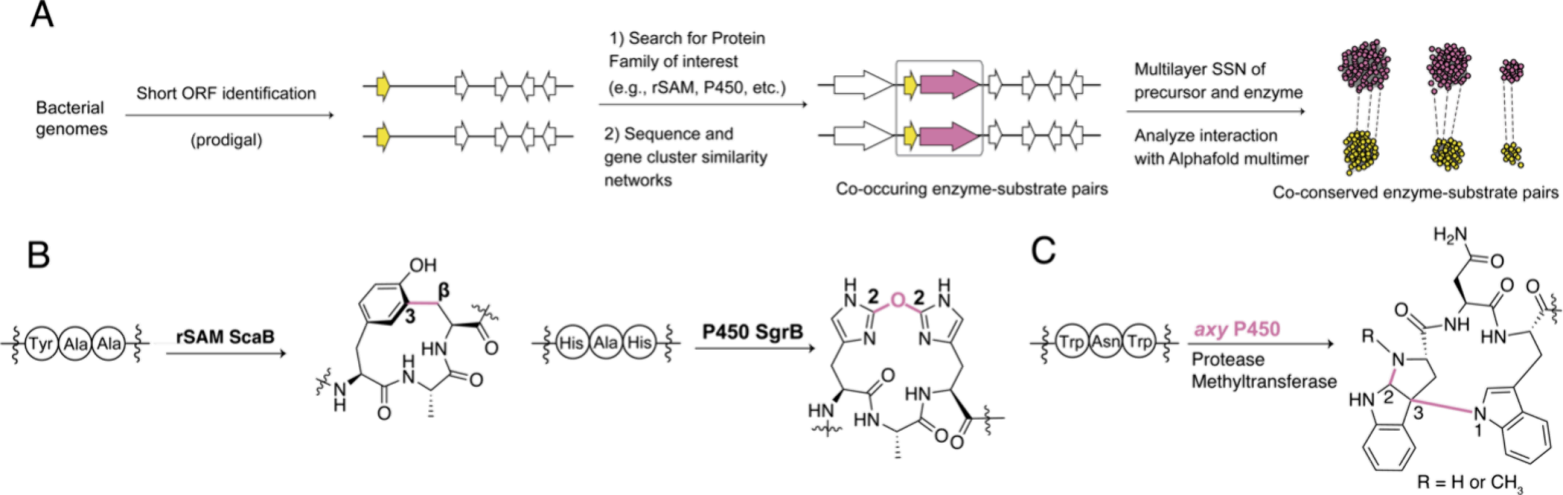
New enzyme chemistry discovered by mining precursor peptides that co-occur with specific protein families. (A) A general description of identifying novel RiPP BGCs through small-peptide and enzyme co-occurrence analysis (SPECO). (B) Novel rSAM- and P450-catalyzed transformations discovered through the SPECO workflow. The natural substrate for ScaB is a His–Ala–Ala motif similar to the substrate for HaaB identified in the triceptide mining studies (Figure 5B). The chemistry depicted in the figure resulted from the mutation of His to Tyr. (C) Novel P450 chemistry identified through a workflow different from SPECO but also utilizing the precursor peptide as the bioinformatic handle.

Homology searches of protein domains have uncovered numerous examples of new enzyme chemistry associated with RiPPs. However, no single protein family is ubiquitous in all RiPP classes. In fact, the only universal feature is the precursor peptide itself, which cannot be reliably retrieved across disparate RiPP classes using any existing tool. Nevertheless, a workflow was developed to identify RiPP BGCs using the precursor peptide (Figure 8A).^130^ First, bacterial genomes were scanned by Prodigal to identify short ORFs, with those being 20–100 amino acids in length being deemed as potential precursor peptides (note: some RiPP precursor peptides are shorter than 20 amino acids, while others are longer than 100 amino acids^27^). Any genes encoding rSAM or P450 enzymes located near the gene of the candidate precursor peptides were analyzed by BIG-SCAPE and CORASON^131^ to facilitate BGC similarity analysis. SSNs for the rSAM enzymes and putative precursor peptides were generated to aid target prioritization. This workflow, termed the small peptide and enzyme co-occurrence analysis (SPECO), facilitated the discovery of a C–C bond between C2 of His and C_β_ of Ala formed by the rSAM enzyme ScaB (WP_184986415.1), a cross-link similar to that discussed above in the triceptide section. Mutation of the His residue in the precursor peptide to a Tyr residue resulted in a linkage between C3 of Tyr_i_ and C_β_ of Ala_i+2_ (Figure 8B).^130^ Subsequent work applied the same workflow to P450s, which revealed a C–O–C ether cross-link between C2 of His_i_ and C2 of His_i+2_ formed by SgrB (WP_051865888.1) (Figure 8B).^132^ The authors also identified the enzymes responsible for forming the C–N cross-link between C7 of Trp_i_ and N1 of Trp_i+3_ in cihunamide^76^ and the rare C–C cross-link between C3 of Tyr_i_ and C5 of Trp_i+2_ observed previously in pseudosporamide.^133^ A peptide–P450 multilayer SSN was constructed to analyze the coconservation of substrate and enzymes, and along with AlphaFold-Multimer substrate–enzyme docking,^64,134^ enhanced the accuracy of precursor peptide prediction. Such analyses may find more novel BGCs going forward, given the enhanced bioinformatic matching of enzymes with their cognate substrates.

Another workflow involving precursor peptide prediction using RBS analysis was utilized to discover new P450-catalyzed transformations.^135,136^ Common RBS motifs were first gathered from 10 bacterial species, followed by compiling genome neighborhoods for all bacterial P450 proteins. Any BGCs containing short peptides encoded downstream of a detected RBS were prioritized. An SSN of the identified putative precursor peptides was constructed, and groups representing conserved and unique precursor peptide sequences were prioritized. This strategy discovered P450s from *Amycolatopsis xylanica* KCTC 19581 (*axy*) (WP_218134608.1), *Streptoalloteichus hindustanus* NRRL B-11280 (SHF91911.1), and *Streptomyces spectabilis* NA07643 (WMW29964.1) that form two C–N bonds: between C2 and the backbone N of a single Trp residue as well as between C3 of the same Trp_i_ and N1 of Trp_i+2_ (Figure 8C).

## IV Outlook

Many RiPPs are macrocyclic structures that possess favorable physiochemical properties such as higher metabolic stability, cell membrane permeability, and reduced entropy cost upon target binding compared to the corresponding linear peptides.^137,138^ Numerous RiPP enzymes possess broad substrate specificity, enabling catalysis on unnatural substrates to accelerate early drug discovery.^27,139−142^ Consequently, libraries of RiPPs have been developed and screened against disease-relevant protein targets, yielding new-to-nature RiPPs as potential drug candidates.^102,143−148^

Besides peptide library construction, RiPP enzymes can also modify larger proteins (e.g., thioamides in methyl coenzyme M reductase,^149^ lanthionines in eukarya,^150^ or ranthionine cross-links in quinohemoprotein amine dehydrogenase^99^). Although many RiPP enzymes require the leader peptide for substrate recognition, impeding their use in non-natural reactions, a workaround involves creating a constitutively active fusion (ConFusion) enzyme by genetically attaching the leader peptide to the enzyme.^151−154^ This strategy circumvented the requirement of a recognition sequence for enzymatic activity and has been exploited to form thiazol(in)es on intact folded proteins.^155^ Hence, together with genetic tools for unnatural amino acid incorporation^156^ and selective chemical modification methods,^157^ RiPP enzymes provide a route to access unnatural scaffolds in proteins for downstream applications. This strategy greatly benefits from the diversity of chemical transformation by RiPP enzymes, which is further amplified by genome and metagenome^158^ mining efforts.

RiPP enzymes also have been shown to be active on small molecules, as exemplified by cyanobactin prenyltransferases.^81^ For instance, the Trp *C*-prenyltransferase KgpF and the Tyr *C*-prenyltransferase LynF processed protected Trp and Tyr derivatives, respectively.^159,160^ Furthermore, LimF was active on cimetidine, a histamine receptor antagonist used to treat duodenal and gastric ulcers.^85^ RiPP enzymes that act on substrates after leader peptidolysis such as the mycofactocin oxidative deaminase MftD (IPR000262, ABK72067.1)^161^ and pyrroloquinoline quinone iron-dependent hydroxylase PqqB (IPR001279, WP_003597599.1) and oxidase PqqC (IPR016084, CAA41581.1)^162,163^ may also be suitable starting points for directed evolution of useful biocatalysts.

In conclusion, this Review showcases recent progress in discovering novel enzymatic activity through genome mining for RiPPs, taking advantage of the relatively easy identification of their genetically encoded substrates. Different mining strategies have been utilized, ranging from searching for pathways similar to characterized RiPPs, especially those containing members of large and functionally diverse enzyme superfamilies, to using more generalized features of RiPP biosynthesis as a biomarker to identify more dissimilar BGCs. These studies have yielded novel enzymatic activity and chemical structures with diverse bioactivities and unique mechanisms of action. The potential application of RiPP enzymes in biocatalysis and bioengineering may contribute to an expanded enzymatic toolbox for the production of new-to-nature compounds.
